# Glances at the Hospitals

**Published:** 1900-11-03

**Authors:** 


### GLANCES AT THE HOSPITALS.

### BRISTOL ROYAL INFIRMARY.

The total ordinary income for 1899 was £11,479, and the total ordinary expenditure was £14,393. At the end of the year 1898 there was a balance of £8,500 due to the treasurer, and at the close of 1899 this had increased to £12,926. This is a very large adverse balance for any charitable institution to incur, and the committee do not think the time suitable to ask for subscriptions to wipe off the debt; but they say that unless relief comes they may before long be compelled to close some of the wards. It is unquestionably the duty of every institution to live within, or at all events not beyond, its income; and if the Bristol public fail to increase the subscriptions, we do not see what other course is open to the committee than to close some of the wards and wipe off the debt by selling some of their investments. The cost of each bed occupied in 1899 was £54 4s., and the cost of each in-patient was £3 13s. 10d. This is lower than it was in 1898, but considerably higher than in 1897. Many improvements and alterations have been in progress during the year, and on these a sum of £6,000 has been spent. At the end of 1898 there were 207 patients in the hospital; 2,880 were admitted during the year 1899, making a total of 3,087. Of this total 2,454 were discharged cured, relieved, or made out-patients, 210 died, 12 were dead when admitted, and 204 remained under treatment. The average duration of treatment in the hospital was 24 days and 8 hours. The death rate, exclusive of those dying within 24 hours of admission, was 5·5 per cent. There were 189 post-mortem examinations. An analysis of the more important medical and surgical cases is given, and separate columns show the "total numbers," the "cured," and the "died." Of pneumonia 71 cases were treated, with 8 deaths; and of enteric fever 65 cases, with 10 deaths. We venture to suggest an extra column for "remarks." What was the immediate cause of death in the 10 fatal cases of enteric? And of the 5 cases of locomotor ataxy put down as cured or relieved, what line of treatment was found best? There were 14 cases of fractured skull, with 3 deaths; and 35 cases of fractured thigh, with 2 deaths. Altogether 1,557 operations were performed. A detailed statement of these, or at least of the 693 major operations, would have been desirable. We are glad to note that an anæsthetic table is given, and from it we learn that chloroform was administered 1,025 times; a mixture of ether and chloroform 98 times; ether 94 times; nitrous oxide and ether 140 times; nitrous oxide 190 times; and cocaine 41 times. For dental cases nitrous oxide was given 1,324 times, making a total of 2,892 administrations of an anæsthetic during one year. The out-patients numbered 39,883. With a grand total of 42,970 patients during the year there is a fine field for medical practice and instruction; and a medical school is attached to the infirmary, in which many scholarships and prizes are offered for competition every year.

### THE CHILDREN'S HOSPITAL, BRADFORD.

The current account shows that the receipts amounted to £1,991, and the total expenditure to £2,440. There was a balance due to the bank of £1,901. On the other hand the investment account shows a balance of nearly £3,000 in favour of the hospital. On January 1st, 1899, the hospital contained 47 patients, and 337 were admitted during the year. Of these 205 were discharged cured, 112 relieved, 7 were incurable, 30 died, and 30 remained in the hospital. The average daily number in the hospital was 40; the average cost per bed occupied was £52 6s., and the average residence was 142 days. The percentage of deaths to the total number discharged was 7·42, including four who died within 24 hours of admission. The out-patients number 1,507 new cases, and represented 5,775 attendances. There were 395 casualties, and 277 remained under treatment The Medical and Surgical tables show the numbers of patients who suffered from various diseases, and, they also give the results of treatment and termination of the cases. Another table shows that 125 operations were performed; but the results of these operations are not given; nor is the anæsthetic used mentioned. A new operating theatre was opened during the year, and it "bears favourable comparison with the theatres in the best institutions of the country." It is stated in the regulations for admission that no letters of recommendation are required the only qualifications being "sickness, poverty and childhood." The first and third of these qualifications are easily enough discovered, but how is the second ascertained? Is there a wage limit of the parent beyond which gratuitous advice is not given?

